# Real-Time Fall Detection in Indoor Environments Using FMCW Radar and Lightweight Ensemble CNN on Edge Devices

**DOI:** 10.3390/s26196189

**Published:** 2026-09-29

**Authors:** Ju-Hong Oh, Young-Joon Park, Seon-In Kim, Ji-Hyoung Kim, Jin-Yong Lee, Yogesh Shankar, Eui-Jong Kim

**Affiliations:** 1 Department of Architectural Engineering, Pukyong National University, Busan 48513, Republic of Korea; juhongoh@pknu.ac.kr; 2 Department of Architectural Engineering, Inha University, Inha-ro 100, Michuhol-gu, Incheon 22212, Republic of Korea; dennispyj@inha.edu; 3 Department of Smart City Engineering, Inha University, Incheon 22212, Republic of Korea; seonin97@inha.edu (S.-I.K.); jhkim19@inha.edu (J.-H.K.); 4 Infineon Asia Pacific Area, Computing & Connectivity Technical Marketing Department, Infineon Technologies Korea, Gyeonggi-do 13595, Republic of Korea; evan.lee@infineon.com; 5 Infineon Asia Pacific Area, Computing & Connectivity Technical Marketing Department, Infineon Technologies Singapore, 8 Kallang Sector, Singapore 349282, Singapore; yogesh.shankar2@infineon.com

**Keywords:** fall detection, frequency-modulated continuous wave radar, edge computing, ensemble CNN, privacy, microcontroller, elderly care

## Abstract

Falls are a leading cause of injury and mortality among the elderly, particularly indoors where privacy concerns limit real-time monitoring. This study developed a privacy-preserving, real-time fall detection system using a frequency-modulated continuous wave radar sensor and a lightweight convolutional neural network (CNN) architecture deployed on an edge device. To address the limitations of wearable and vision-based methods, the system leverages contactless radar sensing and an ensemble of two independent CNN models, namely, a base model designed to capture the entire fall sequence for high recall and a sub-model aimed at pinpointing the fall moment with high precision. The final decision is executed through a voting mechanism that triggers a fall alert only when both models agree, significantly reducing false positives. On 14 recording sessions withheld from the training of both models, the ensemble detected 13 of 14 falls (recall 92.9%, 95% CI 68.5–98.7%) without false alarms, and in a 24-trial on-device test comprising 4 falls and 20 non-fall activities it detected every fall with no false alarm. Running on a low-power microcontroller, the system indicates the feasibility of privacy-preserving on-device fall detection, while validation with older adults and in real living environments remains necessary.

## 1. Introduction

The global elderly population is rapidly increasing, leading to a steady rise in single-person households. Recent reports from international organizations, including the United Nations, project that population aging will accelerate in the 21st century, with the proportion of individuals aged 65 and older expected to more than double by 2050 [1]. Population aging is a pronounced trend across both developed and developing nations, leading to significant transformations in traditional family structures and a marked increase in single-elder households [2]. In key countries such as South Korea, these elderly living alone represent a substantial segment of the senior population. Extended life expectancy in later years has highlighted critical social challenges, including healthcare management, social support, and medical costs for the elderly [3,4]. Older adults living alone often struggle to obtain immediate assistance during emergencies such as falls or acute illnesses, emphasizing the growing necessity for technological aids and comprehensive social interventions to ensure their safety [5].

Falls are particularly common among the elderly, with roughly one-third of individuals aged 65 and older experiencing at least one fall annually [5]. These incidents can lead to not only minor injuries but also heightened mortality, worsening of chronic conditions, and long-term functional decline. In practice, falls often result in fractures, head trauma, and spinal injuries, requiring prolonged recovery periods and significantly hindering daily living activities. Consequently, this increases the demand for long-term care facility admissions and rehabilitation services [6,7]. The social and economic burdens associated with falls are considerable. Falls among older adults impose a substantial strain on national healthcare systems, driven by heightened medical costs, increased caregiving duties for families and society, and substantial productivity losses along with other indirect expenses [8]. Research in developed nations including the United States reveals that annual medical costs associated with elderly falls amount to several billion dollars, with both direct and indirect expenses (e.g., hospitalization, treatment, rehabilitation, and long-term care) steadily rising [9].

To address these societal challenges, a variety of fall detection systems have been developed, primarily classified by their methodologies: wearable devices featuring accelerometers [10,11,12], vision-based techniques [13,14,15], and radar-based technologies [16,17]. Wearable fall detection systems utilize multiple sensors, including accelerometers and gyroscopes, to track user movements in real time, effectively identifying falls while preserving privacy. Nonetheless, the similarity between fall signals and certain activities of daily living (ADLs), such as walking or jumping, can lead to high false-positive rates in these systems [10,11]. Additionally, variations in individual physical characteristics necessitate the customization of threshold values for each user [11]. Practical challenges escalate as systems must support a wider array of activity patterns, resulting in more complex sensor fusion, increased power consumption, diminished computational efficiency, and greater system complexity. Moreover, discomfort from prolonged use and low user adherence have been recognized as obstacles to the widespread adoption of wearable-based systems [12]. The acceptance of these technologies remains notably low among the elderly population.

In contrast, vision-based fall detection systems offer advantages in user convenience and compliance by enabling continuous, non-contact monitoring within indoor environments. Previous studies have proposed methods such as analyzing silhouette changes in video frames [13], extracting motion and spatial features through deep learning techniques [14], and employing optical flow analysis to capture the direction and magnitude of human movement between consecutive frames [15]. However, these approaches face challenges related to performance degradation caused by environmental factors such as lighting variations, reduced resolution, and occlusions. Additionally, convolutional neural network (CNN) models, commonly used for video data analysis, require high-performance computing resources, highlighting the need for further research on real-time processing and model optimization for lightweight applications. Privacy and data protection concerns associated with the use of visual information also present significant barriers to practical implementation.

Radar-based fall detection systems can address the limitations of wearable sensors by enabling non-contact monitoring without compromising privacy and can achieve stable detection regardless of lighting conditions, as they rely solely on movement signal changes via electromagnetic waves. Li et al. [16] proposed a fall detection approach that employs a convolutional bidirectional long short-term memory (CB-LSTM) model, which integrates CNN and bidirectional long short-term memory (BiLSTM), based on spectrogram data acquired from a frequency-modulated continuous wave (FMCW) Doppler radar sensor. In their study, spatial features were extracted using a CNN, while temporal features were analyzed using BiLSTM to improve fall detection performance. Liang et al. [17] presented a fall detection system utilizing 3D point-cloud data from a compact FMCW radar sensor. This approach reconstructs noisy and sparse raw point clouds through a point cloud enhancement (PCE) network and uses the derived pose features as inputs to a lightweight CNN for classifying falls. More recent FMCW and millimeter-wave (mmWave) studies differ mainly in the radar representation and in the network that consumes it. Hsu et al. [18] converted 60 GHz FMCW data into range–Doppler images and cascaded a small trigger CNN with a BiLSTM fall classifier, reporting an accuracy of about 96% over more than 700 recordings collected in three bathrooms and two other indoor spaces. Alhazmi et al. [19] fed point clouds from a 60 GHz mmWave sensor into a PointNet running on an NVIDIA Jetson Nano and classified five activities, including falling, with an accuracy of 99.5%. For rooms with several occupants, Chen et al. [20] tracked the point-cloud center of each person with a single mmWave sensor and validated fall detection with ten participants in ten real-life scenarios, reporting an overall accuracy of 97.9% but a miss rate that increased with the number of occupants.

However, radar-based systems face technological constraints such as reduced real-time responsiveness, increased complexity in signal processing and computation, and higher hardware costs when implemented in architectures that rely on data transmission and central server-based analysis [21]. Specifically, additional research is required to overcome challenges such as distinguishing various activity patterns, reducing signal noise, and avoiding false detections in settings with multiple occupants or complicated indoor layouts. To avoid server based processing, several studies have reduced the computational load of radar-based detection: Ou and Ye [22] shrank a raw-data radar classifier to about 3 K parameters with parallel one-dimensional depthwise convolutions, the trigger model of Hsu et al. [18] has about 2 K parameters, and Tewari et al. [23] combined a low-cost 3.18 GHz continuous-wave radar with a four-stage cascade of classical classifiers designed for low-power hardware, reaching 93.24% accuracy for a single occupant. Hsu et al. observed, however, that false alarms of such lightweight models remained an open issue and therefore added a BiLSTM confirmation stage after the trigger.

Although multiple fall detection methods have been developed, each presents technical challenges, including those pertaining to signal accuracy, real-time performance, user convenience, privacy protection, system efficiency, and adaptability to indoor environments. Most falls occur in private spaces such as bedrooms and bathrooms, where continuous monitoring by external systems is often impractical and, crucially, user privacy must be safeguarded.

In practice, wearable-sensor-based detection methods, such as those employing gyroscopes, offer relatively strong privacy protection by requiring direct attachment to the user’s body. However, these systems inherently struggle to accurately detect falls owing to the similarity between ADL signals and actual falls. Conversely, video-based detection technologies pose significant privacy issues because of the nature of visual data, making their use in residential settings highly problematic. Surveillance technologies in private residences are often restricted by privacy concerns. Therefore, technical solutions that can accurately detect falls in real time without infringing on user privacy are essential.

Swift detection and prompt response after a fall are crucial to minimizing the negative consequences of such incidents. Hong et al. [24] reported that the risk of mortality and severe complications increases significantly if rescue or medical intervention does not occur within 1 h of a fall. In single-occupant elderly households, however, implementing and maintaining centralized systems is frequently difficult, facing challenges such as network failures, latency, and high power consumption [25,26]. Moreover, sensor- and camera-based systems in centralized smart home frameworks inherently risk personal information leakage and the exposure of sensitive data, heightening privacy concerns that limit their effectiveness for fall detection.

Consequently, real-time fall detection systems utilizing edge devices, which can be independently installed in each area without depending on a central server, are emerging as viable alternatives. Edge AI processes data locally without sending information to a central server, thereby enhancing privacy protection, decreasing network load, and improving real-time responsiveness [27,28]. This approach is particularly effective for time-sensitive applications such as fall detection [29] and provides substantial benefits in privacy preservation, as sensitive information remains on the local device and is not transmitted to a central server [30].

Within the current technological and social landscape, research on edge device-based fall detection—minimizing reliance on central servers and ensuring privacy—is advancing rapidly. Hu et al. [31] developed MicroFallNet, an ultralightweight CNN model tailored for edge devices that leverages inertial measurement unit (IMU) sensors integrated into smartwatches. Embedded evaluations revealed a high detection accuracy, averaging above 97%. Fernández-Bermejo et al. [26] presented a real-time fall detection system that combines a Threshold-Based Algorithm (TBA) with a lightweight transformer model using IMU sensors. To conserve the edge device’s computational resources, the transformer neural network was activated only when the sensed acceleration exceeded a predefined threshold. Overall, the proposed model delivered robust detection performance with an accuracy of 95.29%.

Furthermore, research has explored video-based fall detection utilizing edge-acquired data. Lin et al. [32] tackled several drawbacks of traditional camera-based systems, such as high network bandwidth usage, server overload, lack of real-time processing, and privacy issues related to data transmission to servers. They proposed a fall detection system that operates a lightweight deep learning model (YOLO-LW) and a Support Vector Machine (SVM) classifier directly on a compact edge device. Although the model was downsized to approximately 20% of its original size, it achieved detection accuracy metrics—precision, recall, specificity, and F1-score—exceeding 90%. Nonetheless, several challenges remain, such as increased false detections when human body parts are occluded or confused with the background in YOLO-based object detection. Additionally, these models struggle to generalize to complex sequential movements, sudden actions, or atypical behaviors.

Most fall detection studies using edge devices rely on IMU sensors because vision-based models, including those utilizing cameras or radar, demand substantial computational resources and possess intricate architectures. Vision data, typically consisting of three-channel RGB images, expand exponentially with higher resolutions and contain vast information, including color, texture, and background details. Thus, advanced deep-learning models such as ResNet and YOLO are commonly used for object detection. Even with lightweight architectures such as MobileNet or SqueezeNet, the inherent complexity of 2D RGB data remains challenging.

Effective operation in edge environments necessitates the integration of low-power sensors, such as IMUs, with lightweight AI models. However, model compression required for edge deployment must navigate strict constraints on computational resources, including CPU, memory, and battery capacity, which inevitably lead to reduced model complexity and expressiveness. Consequently, a fundamental trade-off arises, as key performance metrics such as accuracy and sensitivity may suffer during this process [33].

Therefore, this study presents a fall detection model utilizing FMCW radar data, enabling the extraction of refined features that focus exclusively on motion-induced signal changes while minimizing the impact of background and irrelevant video information. The model is implemented and evaluated on an edge device to assess its detection performance under resource-constrained conditions. To prevent potential accuracy declines due to limited computational resources, a lightweight ensemble structure is employed, distinguishing the objectives of the base model and sub-model. This approach aims to balance model complexity with detection accuracy. The proposed ensemble runs two compact CNNs with different input windows concurrently on the same microcontroller and declares a fall only when both models agree. This reduces false alarms and improves specificity without adding a heavier confirmation network.

The research questions addressed in this study are as follows:

1. Can a model accurately detect falls during various daily activities using only radar sensors?
2. Can a lightweight fall detection model provide real-time processing and high accuracy on an edge device with limited computational resources?

The remainder of the paper is organized as follows: the development process of the radar-based fall detection model is detailed in Section 2, including the designs of the base and sub-models. The data collection setup using FMCW radar and the labeling strategy is presented in Section 3. The data preprocessing steps and the CNN-based lightweight modeling are presented in Section 4. The performance evaluation results are discussed in Section 5. Finally, the conclusions of this study are summarized in Section 6.

## 2. Development Process

The radar-based fall detection model was developed using data collected from three types of actions: walking, standing up after a fall, and falling. To mitigate overfitting and enhance predictive accuracy, each action was performed 96 times with varying executions, recording 60 s of radar data per trial. In this study, a fall is defined as an event in which the subject falls to the floor and remains lying there without getting up, that is, a fall from which the person cannot recover unaided. Notably, the falling action was broadly defined beyond the moment of impact, encompassing a segment of 15 s from the onset of the fall to the post-fall period when the subject remains stationary. This comprehensive definition better represents real emergency scenarios. Falls followed by an unaided recovery or by continued movement on the floor contain a genuine fall impact but are not treated as alert events. A practical limitation of this definition is that such falls are not reported even when an injury has occurred.

**Figure 1:**
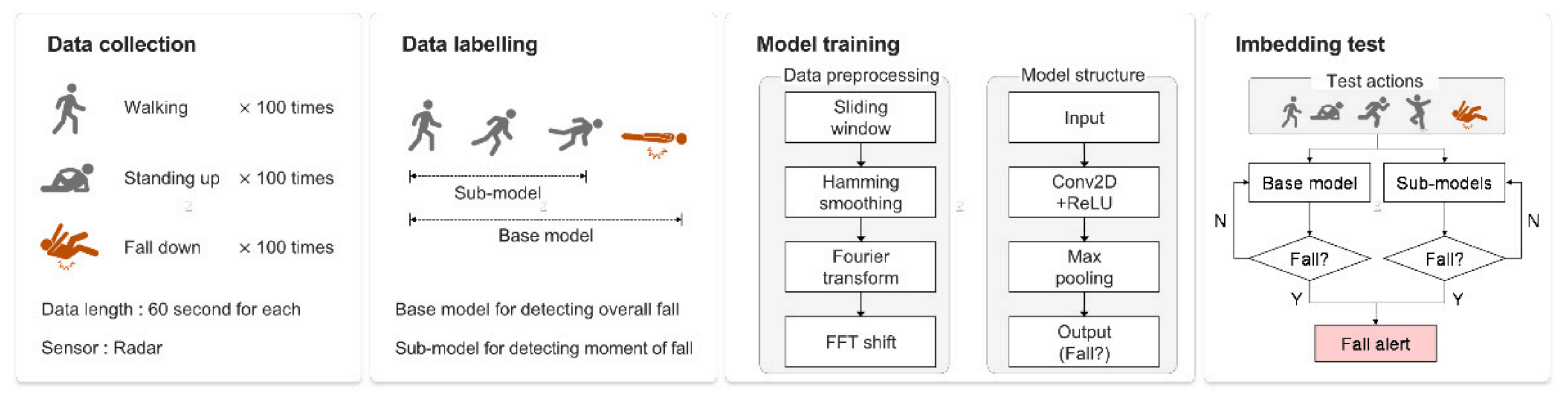
Schematic of the research process.

As illustrated in Figure 1, the research process included several phases: data collection, data labeling, model training, edge device embedding, and final performance evaluation. During the data labeling stage, two distinct labeling schemes were applied to train the base model and sub-model independently. The base model aimed to capture the full context of a fall, with labels covering from the initiation of the fall to the subsequent motionless state. In contrast, the sub-model targeted the precise moment of the fall, labeling only the short interval from the fall’s start to ground contact. This approach enables the model to learn the detailed characteristics of fall events.

For model training, radar data were preprocessed using a sliding window, followed by model-specific windowing, FFT, and channel reordering. The preprocessed data are subsequently fed into a deep learning architecture comprising Conv2D layers, ReLU activation functions, and max-pooling layers for effectively capturing the complex spatiotemporal features of the radar signal.

To minimize missed detections, this study employs an ensemble embedding structure that integrates base and sub-models. Evaluation of the embedded models highlighted the system’s capability to accurately identify actual falls while reducing false positives.

**Figure 2:**
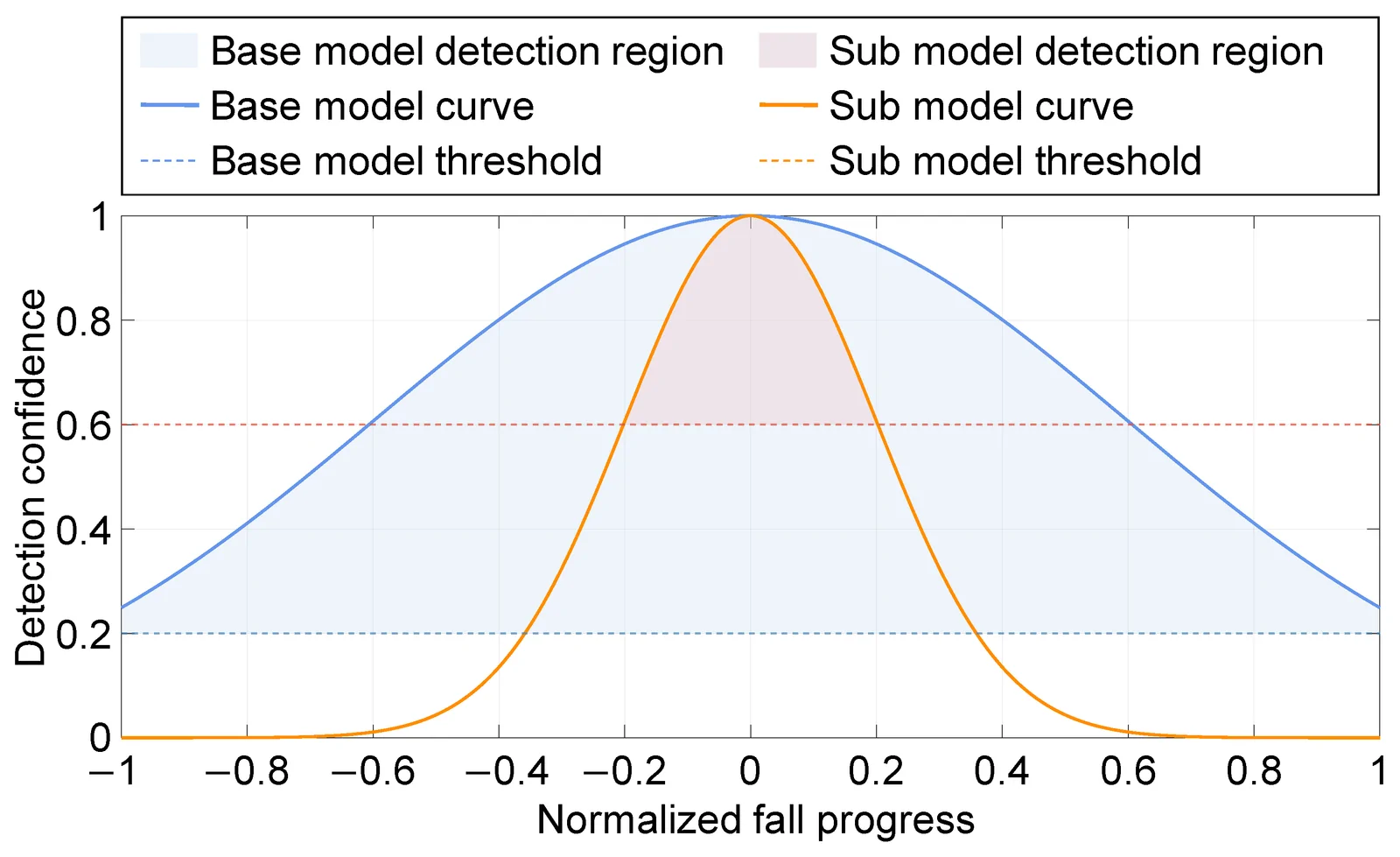
Conceptual comparison of fall detection confidence between the base model and sub-models.

Figure 2 demonstrates the differences in fall detection between the base model and sub-models. The central peak represents the precise fall event. The x-axis depicts a normalized fall progression timeline, where −1 signifies the moment just before the fall begins, 0 indicates ground contact, and +1 denotes the post-fall phase. Each curve illustrates the detection confidence of the model, whereas the dashed line represents the threshold above which the output is classified as a fall. Only regions surpassing the threshold were identified as falls by the model. Figure 2 provides a conceptual illustration of the different detection characteristics of the models, with the curves and threshold shown schematically for clarity.

The base model was designed to prioritize sensitivity, ensuring that no falls were missed. Consequently, a low threshold was set, resulting in a wider detection range and higher sensitivity. However, this also increases the likelihood of misclassifying non-fall actions as falls, thereby reducing precision. In contrast, the sub-model employs a higher threshold to accurately capture only critical fall moments. This approach effectively filters out behaviors that resemble falls but are not actual falls.

The proposed method enhances overall detection performance by leveraging the high recall of the base model with the high precision of the sub-model, thereby achieving an optimal balance between minimizing missed fall events and reducing false detections.

## 3. Experimental Setup

**Figure 3:**
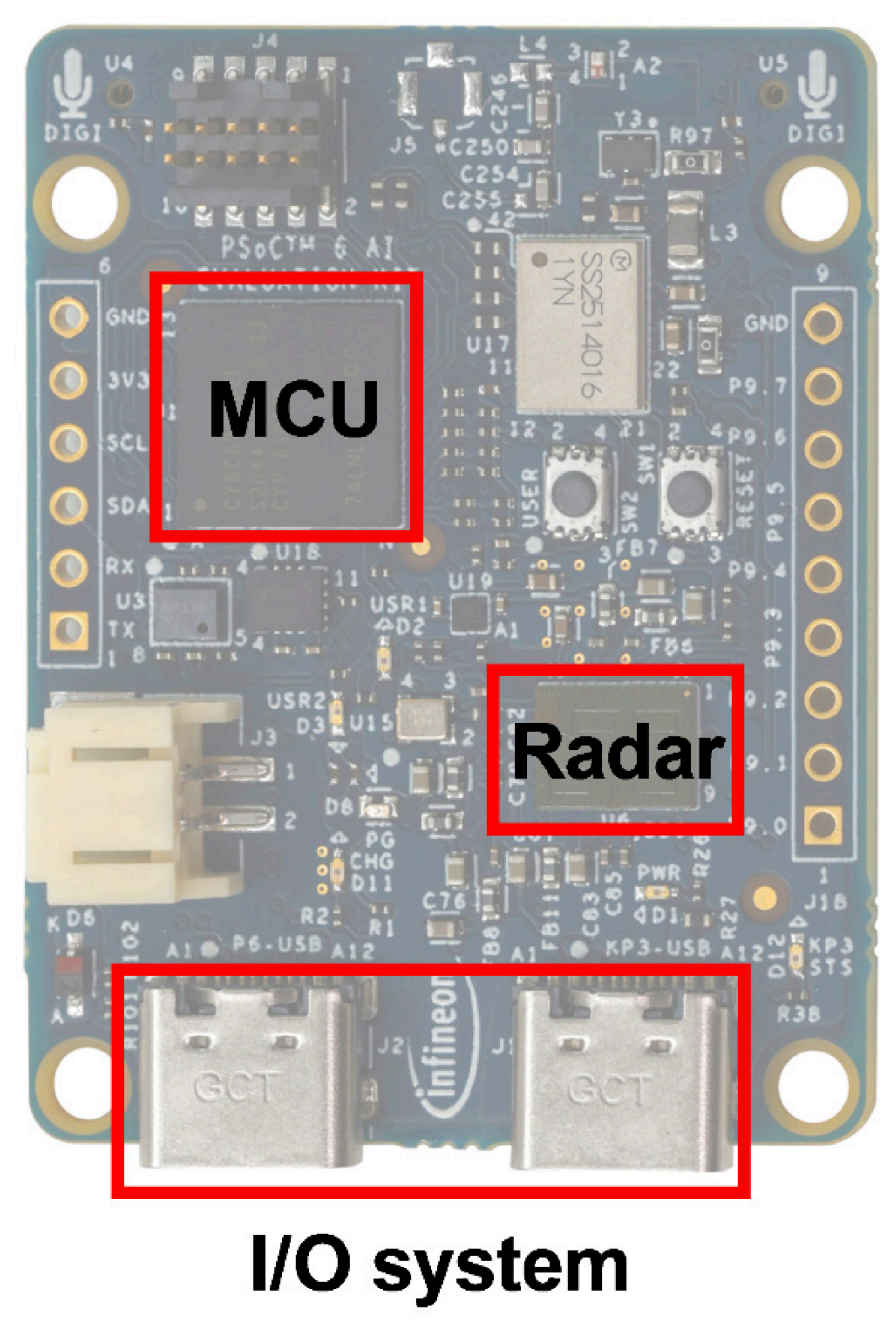
Edge device used in the proposed fall detection model (3.4 cm (W) × 4.4 cm (H)).

For data collection and implementation of the embedded AI model, a low-power edge computing platform based on a dual-core microcontroller was utilized, as illustrated in Figure 3 (Infineon PSOC™ 6 AI Evaluation Kit, Infineon Technologies, Seongnam-si, Republic of Korea). This kit offers a low-power, high-performance platform ideal for real-time edge AI applications. The device features an ultra-low-power dual-core architecture with Arm^®^ Cortex^®^-M4 and Cortex^®^-M0+ microcontrollers, enabling real-time sensor signal processing and efficient on-device deployment and execution of AI models. Furthermore, it is equipped with 512 Mb of external Quad SPI NOR flash memory, allowing both data storage and direct code execution on the device (Execute-in-Place, XIP). Wireless data transmission and remote control or monitoring are also facilitated by the integrated Wi-Fi and Bluetooth combo module [34].

Experimental data were acquired using a 60 GHz FMCW radar system with a 4 GHz bandwidth, which provides sufficient spatial resolution to distinguish human motions. It incorporates two transmitters and four receivers to enable three-dimensional detection of objects along both horizontal and vertical axes. The integrated antenna-in-package design enables seamless system integration without necessitating custom antenna development, thereby enhancing signal processing accuracy and ensuring the reliability of experimental data.

**Figure 4:**
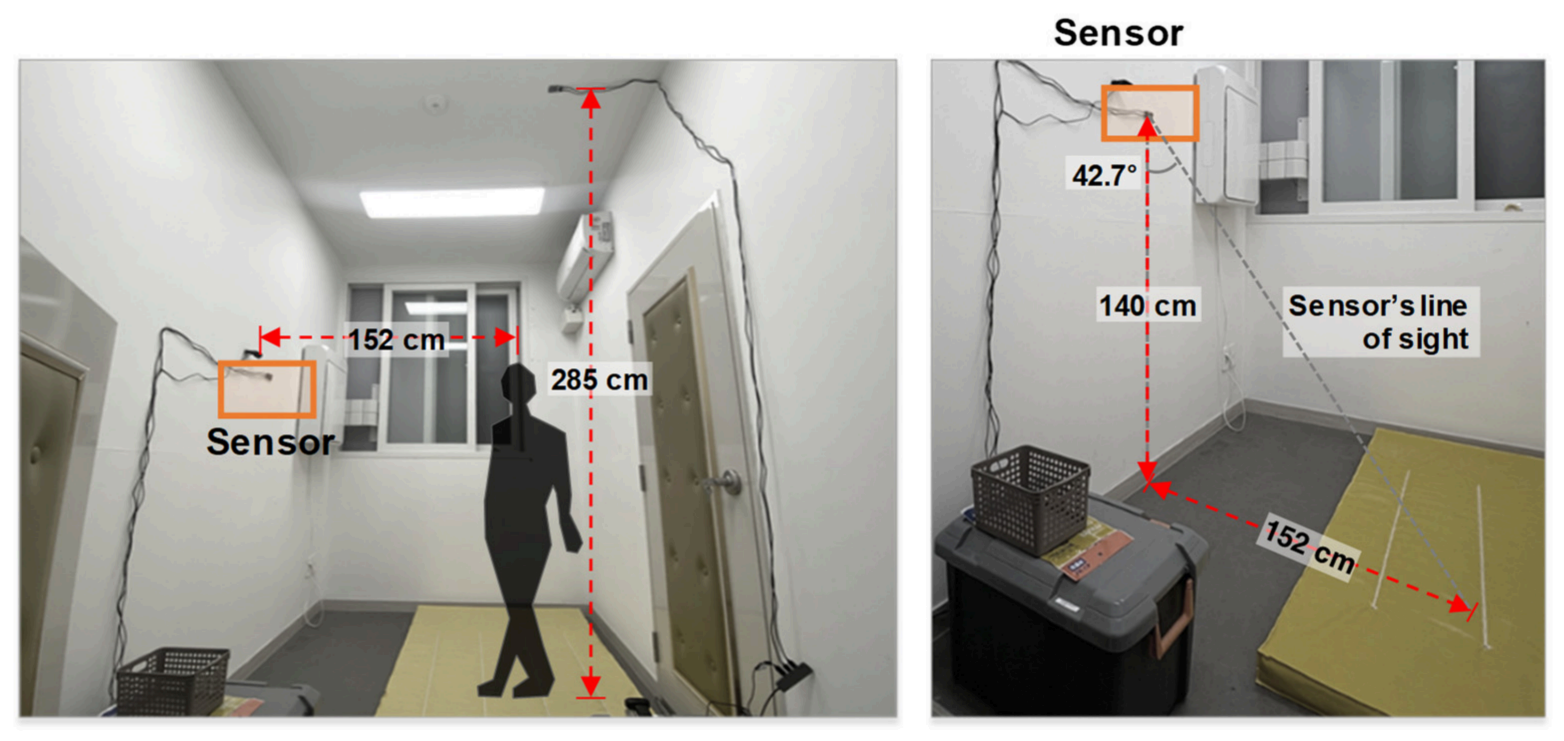
Experimental setup for data acquisition.

The experiment was conducted in an indoor space measuring 2 m in width and 4.5 m in length, with the edge device and other equipment arranged as illustrated in Figure 4. The FMCW radar sensor was mounted on a sidewall at approximately 140 cm above the floor. This installation height was selected to provide the radar sensor with an optimal measurement angle for fall detection, thereby facilitating effective human motion monitoring.

Moreover, the floor and walls of the experimental space were maintained under standard indoor conditions without employing supplementary absorbing materials or treatments typically used to mitigate signal reflection interference. This approach aims to approximate ordinary indoor conditions. The overall experimental setup was designed to provide realistic measurement conditions for evaluating the radar sensor’s signal accuracy.

Radar data were acquired at 0.1 s intervals, producing 600 time-series frames for each 1 min event. Each radar frame was determined by three adjustable parameters: the number of receiving antennas, chirps per frame, and samples per chirp. To achieve high-resolution measurements of distance, velocity, and angle, the system was set to 16 chirps per frame, 64 samples per chirp, and two receiving antennas.

This configuration enhances resolution in three main areas: increasing samples per chirp boosts distance resolution; raising chirps per frame improves Doppler-based velocity resolution; and adding receiving antennas enhances angular resolution. Consequently, each 1 min activity dataset for model training consisted of 600 rows and 2048 columns.

**Figure 5:**
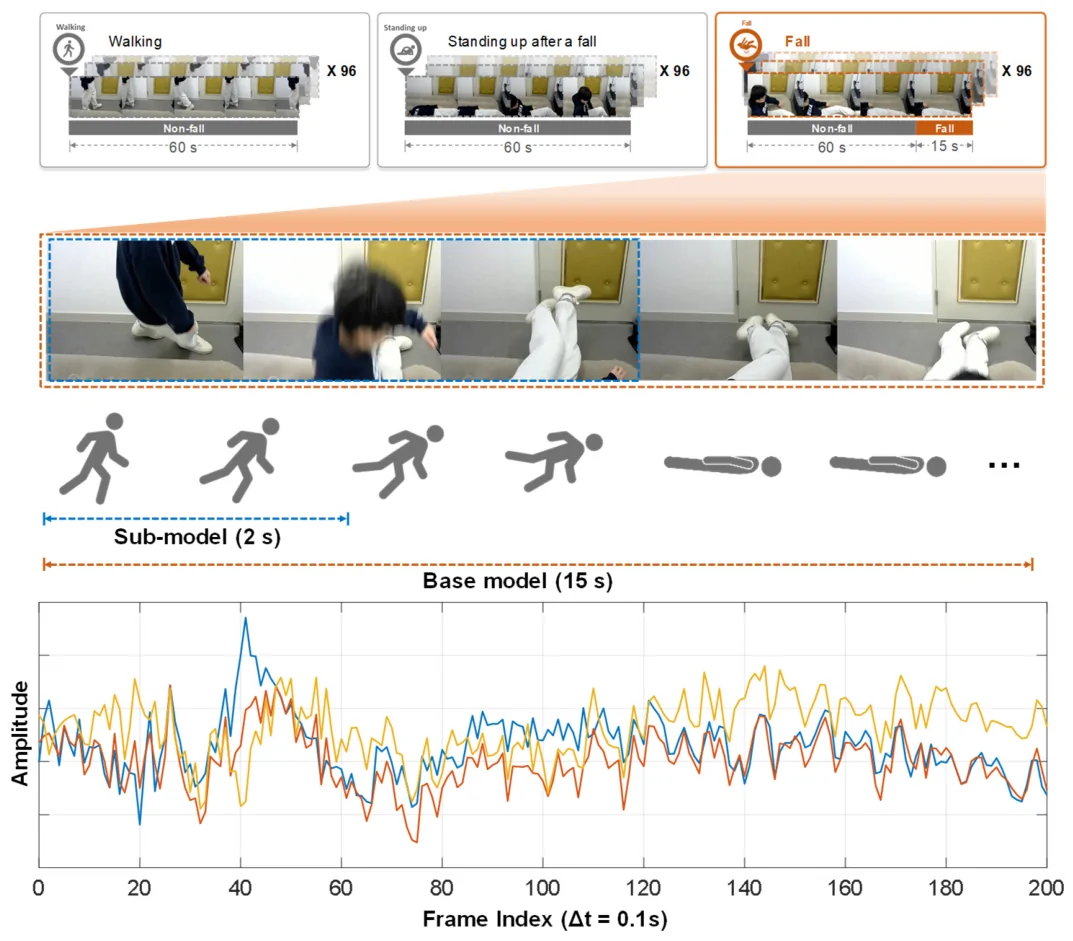
Schematic representation of the labeling process for the base and sub-models.

The radar data labeling process for developing the base and sub-models is illustrated in Figure 5, which displays a subset of frequency components extracted from the 2048-dimensional radar data. During labeling, both video and radar data were utilized to accurately identify the onset of motion and the specific activity performed.

For the base model, a 15 s data segment was labeled as a fall, encompassing the entire sequence from the initiation of the fall event to when the subject was fully on the ground with no further movement. This comprehensive labeling approach aims to capture the full temporal dynamics of a fall event, including the post-fall stabilization phase.

In contrast, the sub-model focuses specifically on the immediate characteristics of falls. Consequently, only a brief 2 s interval, from the initiation of the fall to ground contact, is designated as the labeled fall segment. This method captures the dynamic aspects of a fall, such as initial body movement, acceleration, and the moment of impact, allowing the model to learn more intricate motion patterns.

By separately modeling the overall progression and the immediate moments of a fall through these two labeling strategies, the system is expected to improve detection accuracy by encompassing both macro- and micro-level fall features.

## 4. Methods

### 4.1. Data Preprocessing

**Figure 6:**
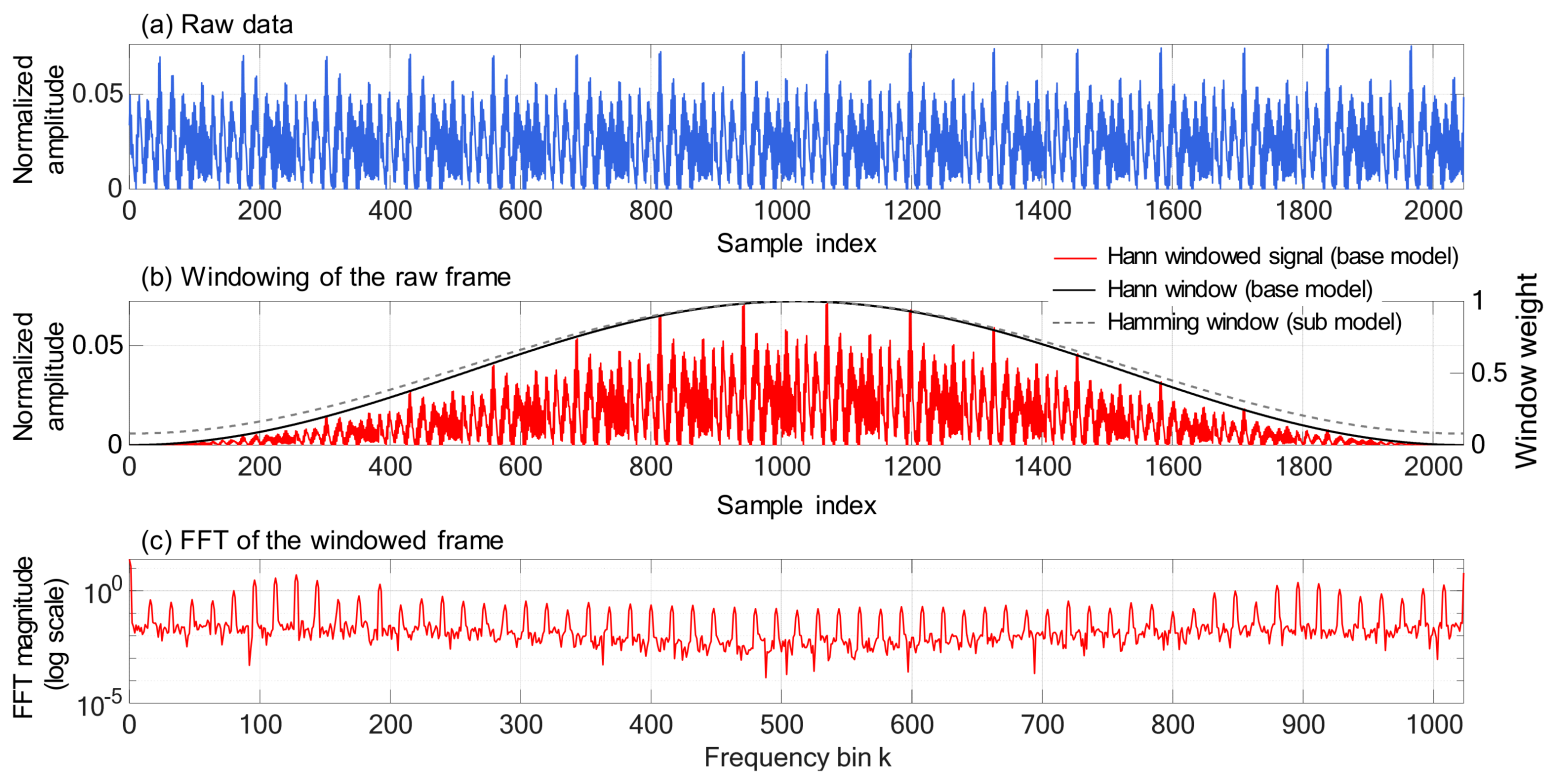
Preprocessing of radar signals for the fall detection models.

To extract meaningful features from the collected radar data, preprocessing was performed as outlined in Figure 6. Initially, a sliding-window approach was adopted to leverage the data’s temporal continuity and provide suitable input for the localization network. For the base model, the window size was set to (50, 2048), comprising 50 consecutive frames corresponding to a 5 s interval, to capture the overall movement context. In contrast, the sub-model utilized a smaller window size of (10, 2048), comprising 10 frames corresponding to 1 s, to enhance sensitivity to rapid motion changes. For both models, the window advances by one frame (0.1 s), resulting in an overlap of 49 out of 50 frames for the base model and 9 out of 10 frames for the sub-model. A window was labeled as a fall only when it lay entirely within the labeled fall segment defined in Figure 5. All other windows, including those partially overlapping the segment, were labeled as non-fall. The same labeling rule was applied to both models.

Subsequently, window smoothing was applied to reduce noise from subtle movements and highlight key signal features and patterns. A Hann window was used for the base model and a Hamming window for the sub-model, because the preprocessing of the two models was configured independently on the development platform. Radar data consists of sinusoidal waveforms featuring a variety of frequency components. To facilitate more efficient analysis, each frame was transformed into the frequency domain using a 2048-point real-valued FFT. This transformation decomposes complex radar signals into their constituent elements—frequency, amplitude, and phase—thereby restructuring the data into a format more conducive to analysis and machine learning.

Finally, an FFT shift block, the last stage of the platform’s preprocessing pipeline, was applied without changing the tensor shape. Through this preprocessing pipeline, the reflected radar signals were refined in both amplitude and frequency characteristics, enhancing fall detection accuracy.

Each radar frame consists of 16 chirps × 64 ADC samples × 2 receiving antennas, flattened in the sensor output order into a 2048-dimensional real-valued vector. Consecutive frames are stacked by the sliding window into a [50, 2048] tensor for the base model and a [10, 2048] tensor for the sub-model. The window function and the 2048-point real FFT are applied along the 2048-sample axis of each frame. The FFT yields 1025 frequency bins, each represented by its real and imaginary parts as two channels, so the tensor after the FFT and the FFT shift is [50, 1025, 2] for the base model and [10, 1025, 2] for the sub-model, which is the input to the CNN.

### 4.2. Fall Detection Modeling

For the binary classification of FMCW radar signals, this study developed a lightweight 2D CNN uniformly applied to both base and sub-models. Depending on the input data’s characteristics, deep learning models are typically built using foundational architectures such as deep neural networks (DNNs), recurrent neural networks (RNNs), or CNNs [35,36]. Specifically, CNNs are highly effective at learning spatial and temporal correlations within data, enabling the extraction of features from both visual and time-series data [37]. They are particularly adept at capturing spatiotemporal features, such as rapid changes in position and velocity, commonly present in radar data [38].

**Figure 7:**
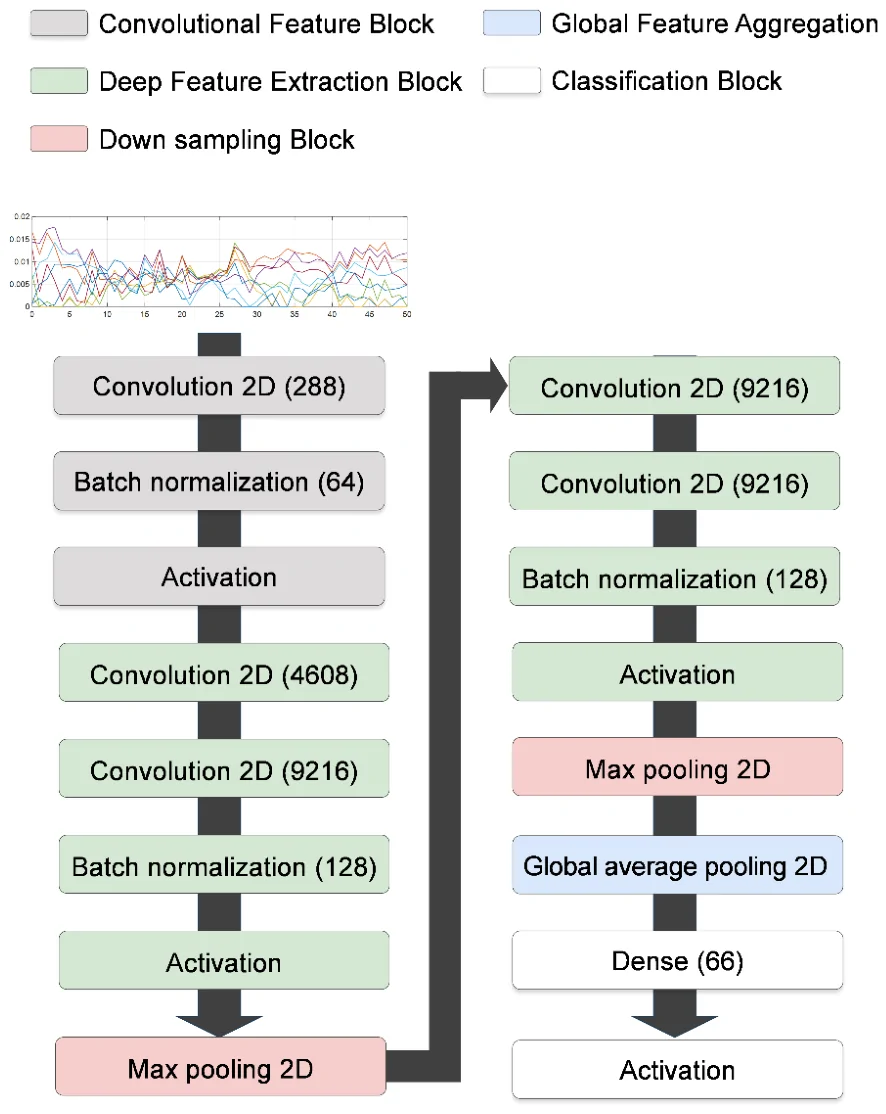
Structure of the proposed base model.

The model architecture employed in this study is presented in Figure 7. In this figure, the numbers in parentheses next to each layer indicate the number of learnable parameters for that layer. The input to the network is the preprocessed tensor described in Section 4.1, [50, 1025, 2] for the base model and [10, 1025, 2] for the sub-model, in which consecutive frames and frequency bins form the temporal and frequency axes, respectively, while the real and imaginary parts of each frequency bin form the two input channels. This representation enables the Conv2D layers to learn temporal patterns across consecutive frames together with relationships among spectral components. The CNN model employed in this study consists of multiple convolutional blocks to process these inputs. Each block includes Conv2D layers with small filter sizes, batch normalization, and ReLU activation functions. Max pooling layers are progressively applied to reduce the spatial dimensions of the data, thereby enhancing computational efficiency.

In the final feature extraction stage, a global average pooling (GAP) layer condenses the high-dimensional feature maps into a one-dimensional vector, minimizing the number of parameters required by the fully connected layer. The final dense layer performs binary classification, and the entire model is designed to maintain a minimal parameter count to ensure optimal performance on edge devices. This architecture is tailored for environments with limited memory and low power consumption, achieving efficient inference and high accuracy. Incorporating batch normalization further enhances training stability while reducing the computational burden.

To develop a lightweight learning model optimized for edge architectures with limited resources, a comprehensive model development environment was utilized. This framework provides an integrated workflow that supports the entire pipeline from data acquisition to deployment on the target device [39]. In this study, the environment facilitated the efficient construction and optimization of the fall detection model. It allows visualization of radar data from the FMCW sensor as interactive graphs and enables manual selection of specific time segments for labeling. This feature improves labeling accuracy, particularly in time-series applications such as fall detection, and enables efficient execution of repetitive labeling tasks.

From a modeling perspective, the platform is designed for lightweight model development tailored to edge device constraints. Users can choose from predefined network templates or create custom model architectures based on their input data format. Parameters such as layer size, activation functions, and pooling methods can be precisely adjusted. In this research, the model was structured as shown in Figure 7, and training hyperparameters—including the number of epochs, batch size, and learning rate—were fine-tuned while monitoring loss and accuracy.

For model training and evaluation, 96 recording sessions, each 60 s in duration, were divided into training, validation, and test datasets. The data split was performed at the recording-session (trial) level. Sliding windows were then generated independently within each session, preventing adjacent or overlapping windows derived from the same session from being distributed across different datasets. The number of sessions and generated windows, along with the class distributions for the training, validation, and test datasets of the base and sub-models, is summarized in Table 1.

The validation dataset was used exclusively for hyperparameter tuning. Because the two models were trained independently, the session composition of the training, validation, and test datasets differed between the models. Accordingly, to ensure an independent and consistent evaluation of the ensemble model, 14 sessions that were not included in either the training or validation datasets of either model were used exclusively for ensemble evaluation. To facilitate deployment on the edge device with limited resources, the trained model was serialized into a lightweight inference format. Subsequently, the model was compiled into optimized C/C++ source code to ensure efficient execution in real time on the target microcontroller. In this study, the trained model was deployed on an edge device, and the fall detection system’s performance was evaluated through 24 trials encompassing eight distinct activities, as presented in Table 2. These eight activities include various daily movements that could easily be mistaken for actual falls, such as sitting down, standing up, lying down, and remaining stationary, as well as dynamic motions (e.g., jumping and running). These activities represent cases that were challenging to classify in previous studies.

## 5. Results

In this section, fall detection performance was evaluated on an event basis. Because a fall is a temporal event that includes motion before and after the actual fall rather than a single instantaneous frame, a temporal tolerance of 2.5 s was applied before and after each window interval labeled as a fall. Consecutive windows classified as falls were grouped into a single predicted event, and a fall event was considered successfully detected when the predicted event overlapped with this temporal tolerance range. The same criterion was applied to all models. Predicted fall events that did not correspond to any actual fall event were considered false alarms.

Performance was evaluated using event-level recall, precision, and false alarm rate. Recall was calculated according to Equation (1) as the proportion of actual fall events that were successfully detected, whereas precision was calculated according to Equation (2) as the proportion of predicted fall events that corresponded to actual fall events. The false alarm rate was calculated according to Equation (3) by dividing the number of false alarm events that did not correspond to actual falls by the total observation time.

During the training phase, different decision thresholds were assigned to the two models according to their respective roles: a relatively low threshold was applied to the base model to reduce missed falls, whereas a relatively high threshold was applied to the sub-model to suppress false alarms. However, for the performance evaluation presented in this section, a fall was identified when the predicted fall probability of each model was equal to or greater than 0.5. In addition, considering the limited number of fall events included in the evaluation, 95% Wilson score confidence intervals were calculated for recall and precision according to Equation (4).

Here, $N_{det}$ denotes the number of successfully detected fall events, $N_{miss}$ denotes the number of missed fall events, $N_{FA}$ denotes the number of false alarm events, and $T_{obs}$ represents the total observation time. In Equation (4), $\hat{p}$ denotes the observed proportion, while k and n represent the number of successful events and the total number of events, respectively. The critical value z of the standard normal distribution was set to 1.96 for a 95% confidence level.

(1) $$Recall=\frac{N_{det}}{N_{det}+N_{miss}}$$

(2) $$Precision=\frac{N_{det}}{N_{det}+N_{FA}}$$

(3) $$False\;alarm\;rate=\frac{N_{FA}}{T_{obs}}$$

(4) $$\hat{p}=\frac{k}{n},\;\;{CI}_{95\%}=\frac{\hat{p}+\frac{z^{2}}{2n}\pm z\sqrt{\frac{\hat{p}(1-\hat{p})}{n}+\frac{z^{2}}{4n^{2}}}}{1+\frac{z^{2}}{n}},\;\;z=1.96$$

### 5.1. Detection Performance and Complementarity of the Two Models

The fall detection algorithm developed in this study utilizes an ensemble voting framework designed for real-time operation on an edge device equipped with an FMCW radar sensor. This architecture consists of two independently developed models with separate objectives: a base model that detects the entire fall sequence and a sub-model that accurately pinpoints the moment of the fall.

The base model aims to identify multiple fall events by analyzing extensive time-series data from the initiation of the fall to the post-fall stabilization phase, making recall the primary performance metric. In contrast, the sub-model focuses on a narrow temporal window around the fall onset to improve precision for accurate fall determination. This complementary design allows the two models to work together, achieving a balanced trade-off between sensitivity and accuracy in fall detection.

**Figure 8:**
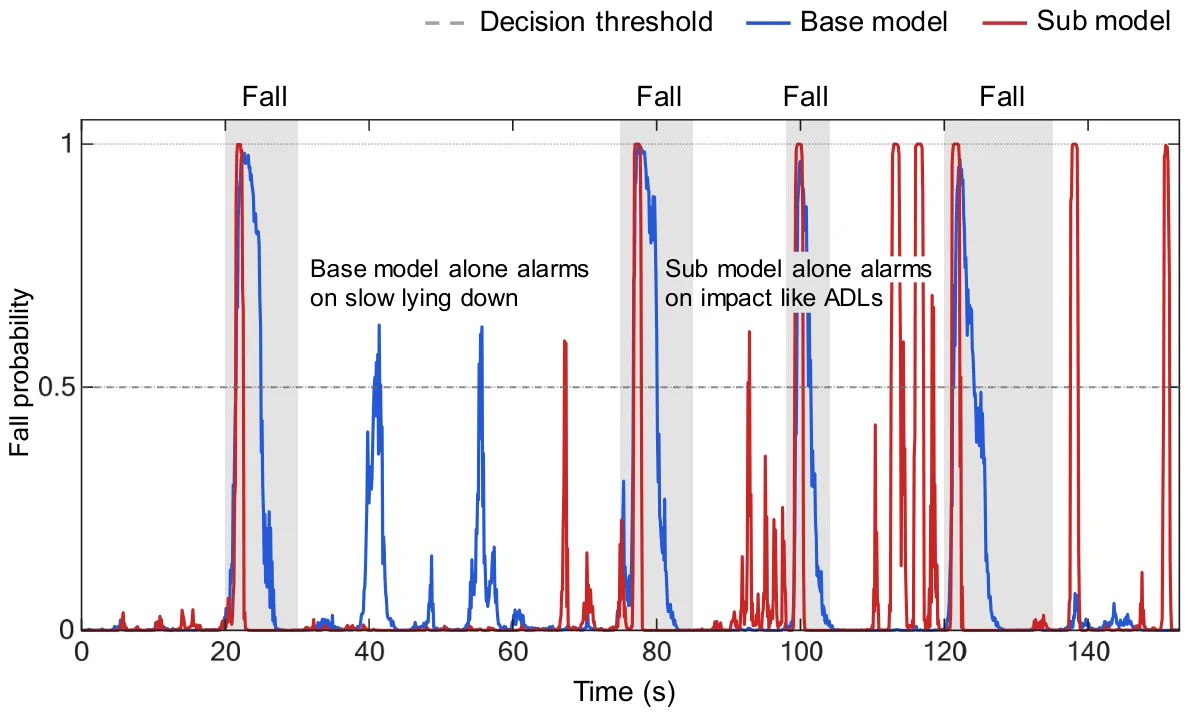
Fall probability of the base model and sub-model.

Figure 8 shows the variation in fall probabilities predicted by the base model and sub-model for the activities listed in Table 2. Among all recorded activities, four actual fall events were included, while the remaining intervals consisted of ADLs such as lying down, jumping, and running. For both models, a time point was classified as a fall when the predicted fall probability was equal to or greater than the decision threshold of 0.5.

Both models produced high fall probabilities during actual fall intervals, demonstrating clear responses to fall events. However, differences were observed in their temporal response characteristics and false-positive behavior for non-fall activities. Because the base model was designed to capture relatively long temporal patterns extending from fall initiation to post-fall stabilization, it exhibited broader and more continuous probability responses during actual fall events. Although this characteristic is advantageous for capturing the overall temporal evolution of a fall, isolated alarms can also occur during certain ADLs involving prolonged postural changes similar to those of falls, such as lying down and staying down. Thus, the base model may generate false-positive responses to non-fall activities exhibiting temporal patterns similar to those of falls.

In contrast, the sub-model was designed to capture rapid changes occurring around the moment of a fall and therefore exhibited shorter and more distinct responses than the base model during actual fall events. However, isolated alarms can also occur during ADLs involving abrupt impacts or rapid body movements, such as running, jumping, standing up after a fall, and falling and rolling. Accordingly, the sub-model may generate false-positive responses when non-fall activities exhibit transient impact-related patterns similar to those occurring during falls.

Overall, the base model and sub-model respond to different temporal characteristics and exhibit distinct false-positive patterns for non-fall activities. Therefore, the complementary characteristics of the two models were utilized to suppress isolated false alarms occurring during non-fall conditions while enabling actual fall events, to which both models respond concurrently, to be identified more reliably.

To quantitatively evaluate the effect of combining the two models on fall detection performance, their performance was compared under identical conditions using 14 sessions that were not included in the training datasets of either model. As shown in Table 3, the base model detected all 14 fall events but generated 38 false alarms, resulting in a precision of 26.9%. In contrast, the sub-model also detected all 14 fall events without generating any false alarms. When the combination rule was applied, in which a fall was identified only when both models detected a fall concurrently, 13 of the 14 fall events were detected with no false alarms, corresponding to a recall of 92.9% (95% confidence interval, 68.5–98.7%).

### 5.2. Embedding Test Results

To assess the performance of the model on an edge device and quantitatively demonstrate that edge environment constraints are effectively addressed, it is essential to evaluate not only prediction accuracy but also metrics such as model compactness, real-time responsiveness, and energy efficiency. When deploying models on edge devices, assessing lightweight design, real-time processing capability, and energy consumption under limited resources is crucial.

**Figure 9:**
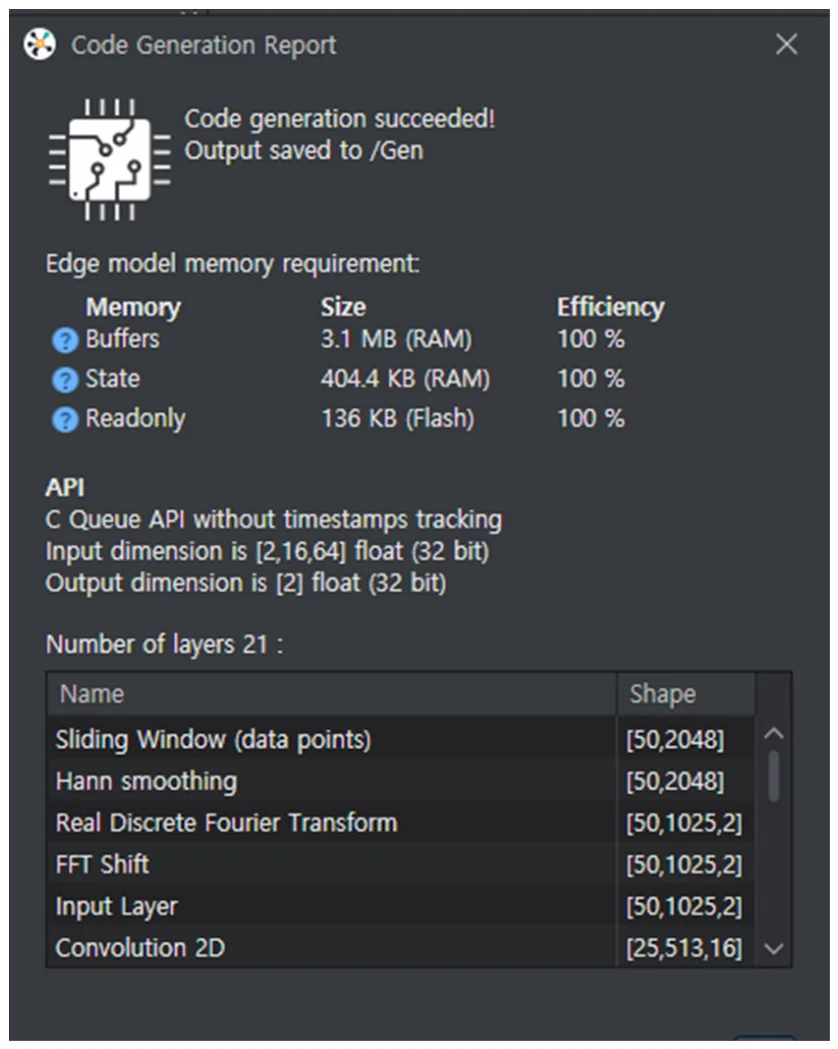
Code generation report for the edge deployable base model.

The structure of the base model is shown in Figure 7, and its code generation report is shown in Figure 9. The base model shown in Figure 7 has 32,930 learnable parameters. The sub-model follows the same block design with a shorter input window and a different filter configuration and has 19,042 parameters. The ensemble therefore comprises 51,972 parameters in total, which is two to three orders of magnitude fewer than conventional large-scale CNN models such as ResNet-50 (~25.6 million parameters) and MobileNetV2 (~3.4 million parameters) [40,41]. As shown in Figure 9, the code generation report from the development platform indicates that the floating-point implementation of the base model requires 136 KB of flash memory for the model weights and 3.5 MB of RAM for buffers and state variables; the corresponding report for the sub-model indicates 82 KB of flash memory and 0.9 MB of RAM.

In this study, real-time fall detection was performed through continuous inference on each radar frame. Both models were configured to produce a classification output for every new radar frame (0.1 s) using the most recent sequence of frames within their respective input windows. An ensemble alert was issued when both the base model, using a 5 s input window, and the sub-model, using a 1 s input window, classified the current input as a fall. Accordingly, the timing of the alert was primarily determined by the 5 s input window of the base model.

In standard training environments without quantization, most deep learning frameworks utilize the float32 format, which consumes approximately four bytes of memory per parameter. As a result, even when employing identical model architectures, memory consumption is more than four times greater than that of an int8-based quantized model. This inefficiency poses challenges in environments where model compactness is crucial.

**Figure 10:**
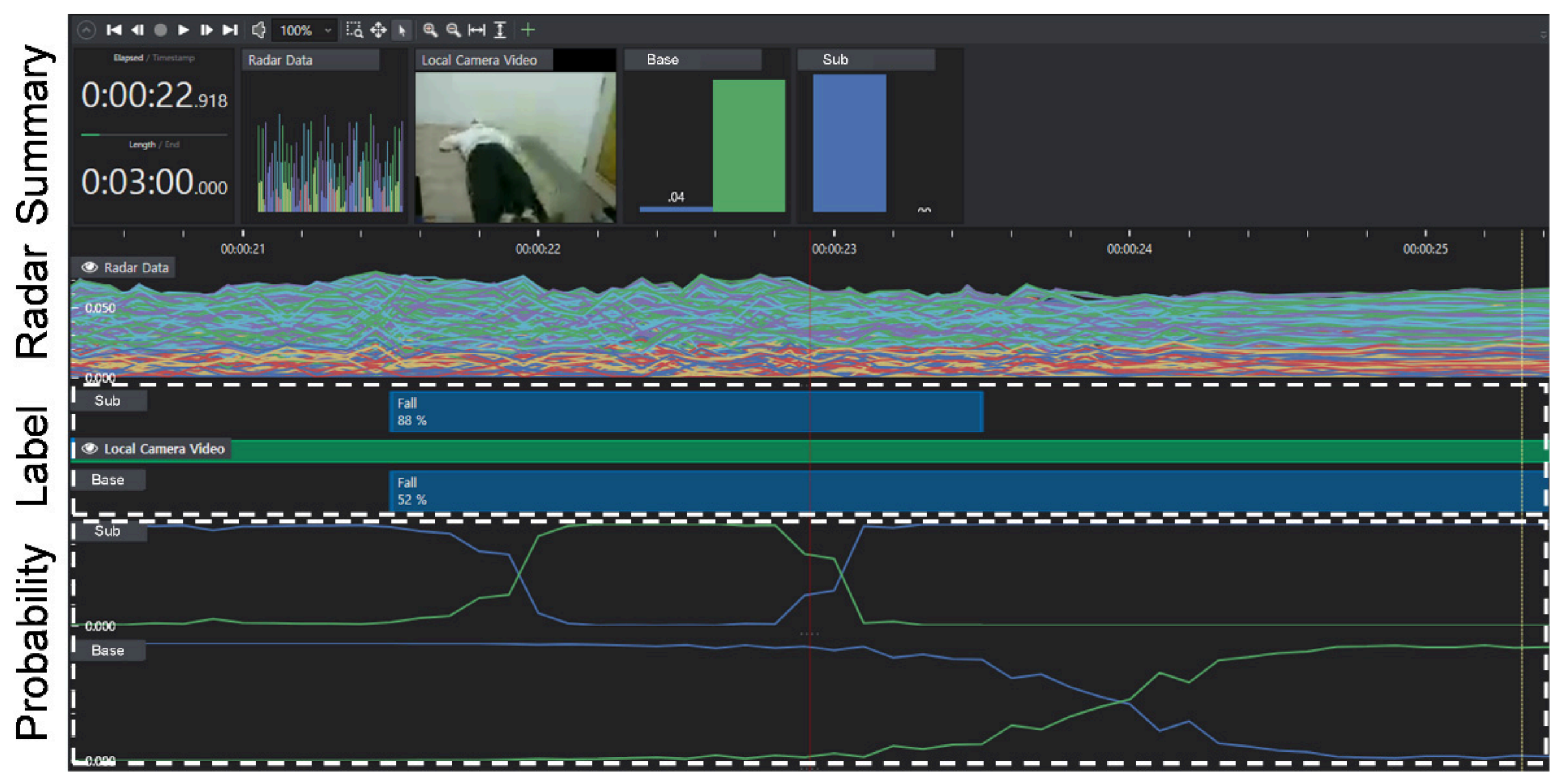
Visualization of real-time fall detection results on the edge device using the ensemble model.

To evaluate the fall-detection capabilities of the trained ensemble model under realistic conditions, the model was deployed on an actual edge device. The system was assessed using a real-time monitoring interface, as illustrated in Figure 10, which facilitates the concurrent visualization of real-time video, radar data, and classification outputs from both the base and sub-models. The display interface consists of four main sections: a summary section at the top providing a real-time overview of ongoing operations; a radar data section in the center showing time-series radar signals; a label section at the bottom displaying classification results from the base and sub-models; and a probability panel presenting confidence scores for fall detection from each model.

Table 4 presents the results of embedding tests performed across eight activities, including scenarios that closely resemble falls. These eight activities encompass a range of daily movements that can easily be mistaken for actual falls, including walking, standing up, running, jumping, lying down and remaining down, standing up after a fall, and falling and rolling, alongside the genuine fall event to be detected—specifically “falling and staying down.” During the evaluation, an ensemble strategy was employed, confirming a fall event only when both the base model and sub-model simultaneously identified it as such. As listed in Table 4, all four actual fall events (“falling and staying down”) were detected without any false alarms.

Notably, in the base model, two instances of the “lying down and staying down” activity—which are common in daily life and do not indicate actual falls—were incorrectly classified as falls. This misclassification likely resulted from the model confusing a static lying posture with a post-fall-stabilized state. However, the sub-model accurately identified these activities as non-falls, thereby preventing false detections in the final ensemble decision. These findings demonstrate that the ensemble strategy maintains high accuracy and a low false alarm rate, even when various daily activities resembling real falls are included.

Conversely, the sub-model occasionally misclassified events such as “running,” “jumping,” “falling & standing up,” and “falling & rolling” as falls. This occurred because the sub-model was specifically designed to be highly sensitive to abrupt motions over brief periods. Consequently, its detection accurately identifies actual fall-related events such as “Falling & Standing up” and “Falling & Rolling.” Nonetheless, it has limitations, misclassifying high-speed vertical movements such as “Running” and “Jumping” as falls. These false detections were effectively mitigated by the base model, which correctly recognized these actions as non-fall events.

**Figure 11:**
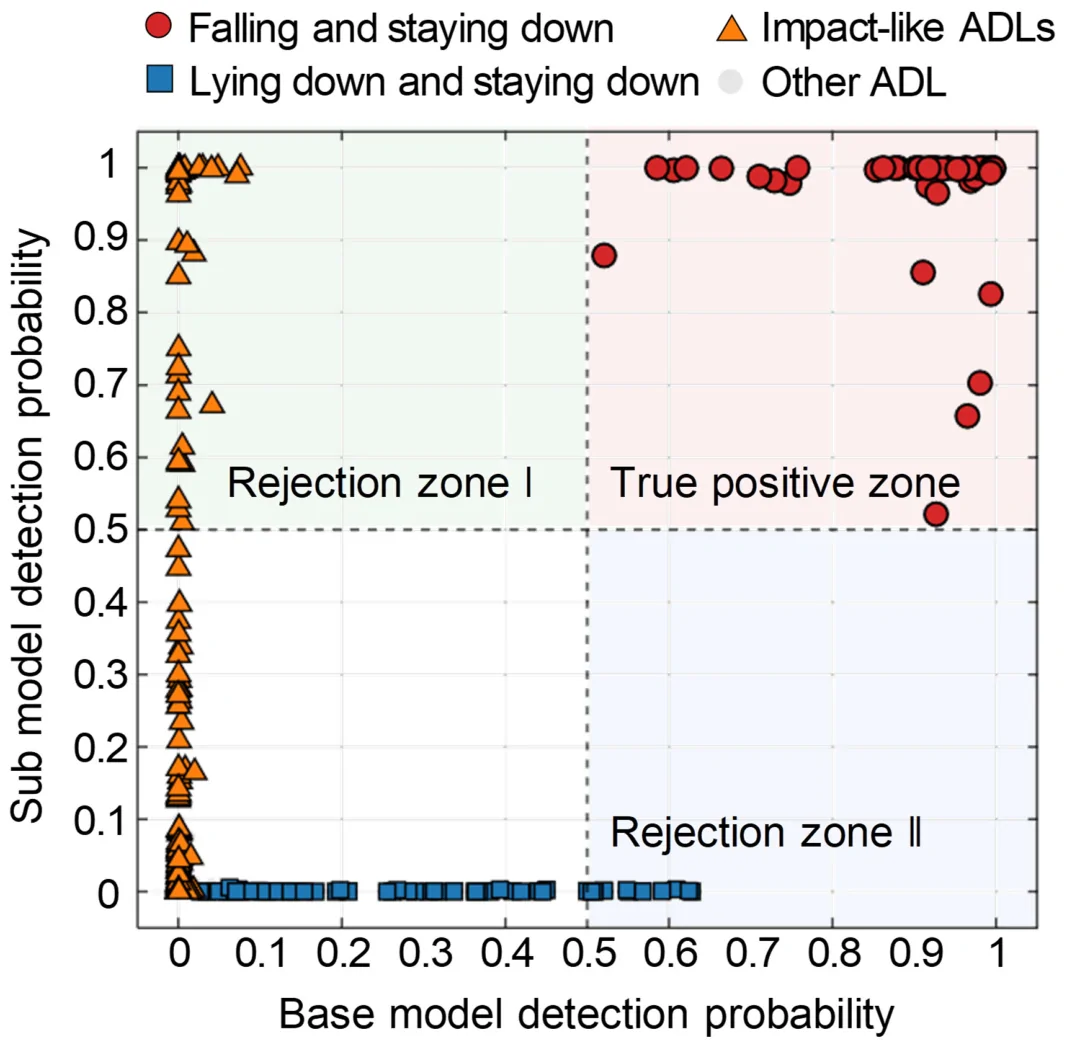
Distribution of falls and confounding ADLs in the 2D decision space formed by the prediction probabilities of the base model and sub-model.

The base model sustains high sensitivity in detecting falls by utilizing a broader time window, while the sub-model complements it by enhancing precision for abrupt, dynamic motion changes. To verify the distinct roles of each model in differentiating falls from ADLs, Figure 11 presents an analysis of the 2D decision space constructed using the fall prediction probabilities of each sub-network during the embedding test. The figure illustrates four quadrants defined by a decision threshold of 0.5, visually demonstrating the system’s logical filtering mechanism. The 0.5 boundary shown here is the default decision rule of the deployed system, applied equally to both models for visualization, and is distinct from the model-specific thresholds used in the training phase.

First, actual “falling and staying down” data were clustered in the first quadrant (True positive zone), where both models recorded high probabilities. This indicates physical consensus: the sub-model captures the rapid impact signal upon falling, while the base model simultaneously recognizes post-fall motion dynamics.

Second, impact-like ADLs involving rapid body movements, such as “running,” “jumping,” “standing up after a fall,” and “falling and rolling,” temporarily increased the sub-model’s probability, in some cases to over 0.9. However, these events were isolated in the second quadrant (Rejection Zone I) because the base model, leveraging an extended temporal receptive field, identified the context before and after the impact and correctly classified them as non-falls. Third, “lying down and staying down” poses a risk of triggering false positives in the base model due to the similarity of the final posture to a post-fall state. However, these instances were classified into the fourth quadrant (Rejection Zone II), as the sub-model detected the absence of impact energy characteristic of falls, thereby maintaining a low probability.

Consequently, ambiguous situations that could lead to false alarms when relying on a single model were confirmed to be effectively rejected by the proposed logical ensemble structure. Across the predefined scenarios, the conjunctive ensemble did not misclassify any non-fall activities that triggered only one of the two models as falls. These results demonstrate that combining the base and sub-models effectively reduced false alarms associated with non-fall activities.

## 6. Conclusions

This study developed a real-time fall-detection framework that integrates FMCW radar sensing with a lightweight ensemble CNN model optimized for edge devices. The proposed system provides an alternative to existing wearable and vision-based solutions by offering nonintrusive monitoring with robust privacy protection. By partitioning the fall detection task into two complementary objectives—comprehensive sequence detection and precise fall moment recognition—the ensemble structure effectively balances recall and precision. When evaluated with various daily activities resembling falls in indoor settings, the system detected all scripted falls without false alarms under the tested conditions.

Furthermore, the model was successfully deployed on an ultralow-power microcontroller unit (MCU). Radar-based sensing combined with on-device inference eliminates latency and data transmission issues, making the solution scalable and privacy-compliant.

This study addresses three key gaps in the field. First, an indoor sensing framework is introduced to capture spatial information using a single sensor while preserving user privacy. Second, a dual-model ensemble approach is implemented, in which the base and sub-models are separated to complement each other, thereby suppressing the false alarms produced by each model independently. Third, on-device operation is demonstrated across scripted activities, including walking, jumping, and running.

Despite its potential, this study has several limitations. The dataset was collected from only three participants, did not include elderly subjects, and the system was not evaluated in multi-person environments. In addition, the evaluation on 14 sessions not used for model training comprises 14 fall events recorded, so the reported detection rates carry wide confidence intervals, and the absence of false alarms bounds the false-alarm rate only to below approximately 14 per hour at the 95% confidence level. The 24-trial on-device test likewise contains only four falls. These limitations restrict the generalizability of the present findings. Future work will therefore include a larger and more diverse participant population, with particular emphasis on elderly subjects, and will evaluate the robustness of the system in multi-person and elderly care environments. The proposed approach should also be compared with external lightweight baseline models under identical evaluation conditions to further clarify the contribution of the dual-window ensemble strategy. Further efforts will also focus on extending the system to a wider range of fall types and improving efficiency through hardware–software co-design. Ultimately, the proposed system offers a viable and scalable method for contactless activity recognition and fall detection in smart healthcare environments.

## Figures and Tables

**Table 1:** Data distribution across training, validation, and test sets.

Model	Dataset	No. of Sessions	No. of Windows	Fall Ratio (%)
Base model	Training	58	32,567	17.3
	Validation	19	10,634	17.3
	Test	19	10,592	17.9
Sub model	Training	57	34,302	2.0
	Validation	19	11,358	2.0
	Test	20	11,973	2.0

**Table 2:** Embedding test scenarios.

Scenario	Description	Number of Tests	Fall Event
Walking	A natural movement of moving forward at a steady pace using both feet alternately	2	No
Standing up	The act of raising the body from a sitting or lying position to an upright position	5	No
Running	A faster movement than walking in which both feet alternately push off the ground in rapid succession	2	No
Jumping	Propelling the body into the air by forcefully pushing off the ground with one or both feet	5	No
Lying down and staying down	Slowly moving into a lying or prone position and staying still	2	No
Standing up after a fall	Falling to the ground and then getting back up	2	No
Falling and rolling	After falling, the body rolls or tumbles while lying down	2	No
Falling and staying down	Falling and staying on the ground without getting up	4	Yes

**Table 3:** Fall detection performance of individual and combined model configurations.

Model Configuration	Falls	Detected	Missed	False Alarms	Recall	Precision
Base model	14	14	0	38	100	26.9
Sub model	14	14	0	0	100	100
Ensemble model	14	13	1	0	92.9% (95% CI, 68.5–98.7%)	100

**Table 4:** Embedding test results.

Scenario	Number of Trials	Number of Fall Detection			Fall Event
		Base	Sub	Ensemble	
Walking	2	0	0	N	No
Standing up	5	0	0	N	No
Running	2	0	1	N	No
Jumping	5	0	2	N	No
Lying down and staying down	2	2	0	N	No
Standing up after a fall	2	0	2	N	No
Falling and rolling	2	0	2	N	No
Falling and staying down	4	4	4	Y	Yes

## Data Availability

The raw data supporting the conclusions of this article will be made available by the authors on request.
